# The Development of a Standardized Quality Assessment Material to Support Xpert^®^ HIV-1 Viral Load Testing for ART Monitoring in South Africa

**DOI:** 10.3390/diagnostics11020160

**Published:** 2021-01-22

**Authors:** Lara Dominique Noble, Lesley Erica Scott, Asiashu Bongwe, Pedro Da Silva, Wendy Susan Stevens

**Affiliations:** 1 Department of Molecular Medicine and Haematology, School of Pathology, Faculty of Health Sciences, University of the Witwatersrand, Johannesburg 2000, Gauteng, South Africa; lesley.scott@wits.ac.za (L.E.S.); wendy.stevens@wits.ac.za (W.S.S.); 2 National Priority Program, National Health Laboratory Service, Johannesburg 2000, Gauteng, South Africa; asiashu.bongwe@nhls.ac.za (A.B.); pedro.dasilva@nhls.ac.za (P.D.S.)

**Keywords:** HIV viral load, external quality assessment, verification, quality, thermostable, PrimeStore MTM0

## Abstract

The tiered laboratory framework for human immunodeficiency virus (HIV) viral load monitoring accommodates a range of HIV viral load testing platforms, with quality assessment critical to ensure quality patient testing. HIV plasma viral load testing is challenged by the instability of viral RNA. An approach using an RNA stabilizing buffer is described for the Xpert^®^ HIV-1 Viral Load (Cepheid) assay and was tested in remote laboratories in South Africa. Plasma panels with known HIV viral titres were prepared in PrimeStore molecular transport medium for per-module verification and per-instrument external quality assessment. The panels were transported at ambient temperatures to 13 testing laboratories during 2017 and 2018, tested according to standard procedures and uploaded to a web portal for analysis. A total of 275 quality assessment specimens (57 verification panels and two EQA cycles) were tested. All participating laboratories met study verification criteria (*n* = 171 specimens) with an overall concordance correlation coefficient (ρ_c_) of 0.997 (95% confidence interval (CI): 0.996 to 0.998) and a mean bias of −0.019 log copies per milliliter (cp/mL) (95% CI: −0.044 to 0.063). The overall EQA ρ_c_ (*n* = 104 specimens) was 0.999 (95% CI: 0.998 to 0.999), with a mean bias of 0.03 log cp/mL (95% CI: 0.02 to 0.05). These panels are suitable for use in quality monitoring of Xpert^®^ HIV-1 VL and are applicable to laboratories in remote settings.

## 1. Introduction

Several countries striving to attain their 2020 UNAIDS 90%/90%/90% targets for global HIV healthcare [1] struggle with the third 90% (virological suppression). Fast-track targets were designed to address this [2], aiming to increase the number of people living with HIV (PLWH) accessing treatment and achieving virological suppression. Current global estimates show that 25.4 million people, approximately 67% of PLWH, were accessing antiretroviral therapy (ART) by end-2019 [3], and monitoring needs are likely to increase over the next decade as more people access ART. A total of 5,231,809 (70%) patients currently access ART in South Africa alone [4], with the number expected to increase as the remaining PLWH are reached. The recommended test for monitoring ART response is HIV viral load (VL) quantification [5]. This has historically been performed at centralized laboratories owing to the number of specimens requiring processing, the logistical needs of the available technologies, and the lack of accurate and cost-effective near patient VL technologies. South Africa has addressed the VL scale-up testing needs through a highly centralized model within the National Health Laboratory Service (NHLS), which is responsible for laboratory testing of ~80% of the population. The capacity of the 16 high throughput, centralized HIV VL laboratories has been further augmented through automation and instruments with increased throughput [6,7,8,9,10,11,12,13,14], most recently the cobas^®^ 8800 (Roche Molecular, Pleasanton, CA, USA) and Alinity-*m* (Abbott Molecular, Des Plaines, IL, USA) systems.

Nonetheless, there are a number of PLWH who live in remote areas and whom are unable to access the centralized facilities, as highlighted during the current COVID-19 pandemic, either because no collection facilities exist within travelling distance or because specimen transport to the testing laboratories is limited by the stability of HIV RNA plasma [15,16]. While studies showing long-term stability of HIV in whole blood are available [17,18,19], the manufacturers of the VL technologies recommend testing within 24 h, with separation of plasma within six hours and specimen refrigeration [20,21], primarily to maintain the quality of low VL specimens and to overcome the extreme temperatures (>30 °C) in many high HIV prevalence regions. The use of plasma preparation tubes (PPT; Becton Dickinson, Franklin Lakes, NJ, USA) was introduced [19,22,23] to increase the specimen transport window to at least 24 h [24,25], although specimens should still be separated within six hours of collection and prior to transport [19]. Alternative options to plasma-based testing include the use of dried blood spots (DBS) and several countries have shown that this is a feasible option for remote collection and centralized testing [26,27,28,29,30,31]. The DBS matrix is nonetheless challenged by inaccuracies at the clinically relevant range (1000 copies per milliliter (cp/mL)) as the VL at this threshold increases due to the contribution of cell-associated RNA [32]. While this remains the recommended threshold for virological failure [5], there is contention regarding the use of DBS at VL below 5000 cp/mL [33,34]. A decentralized model, utilizing mobile or remote clinics, may address the needs of PLWH in remote areas through a tiered laboratory network [15,16,35], similar to that originally used for CD4 scale-up [36]. As such, the NHLS National Priority Program (NPP), in collaboration with the South African Department of Health, and through the Global Fund to Fight HIV, Tuberculosis and Malaria (Global Fund; Geneva, Switzerland), performed a pilot evaluation of the Xpert^®^ HIV-1 VL (Cepheid, Sunnyvale, CA, USA) in remote district laboratories. The Xpert^®^ HIV-1 VL assay was previously evaluated in collaboration with the NPP [37] and received World Health Organisation pre-qualification status in 2017 [38]. In addition to being one of the few commercially available POCT HIV VL assays ready for implementation at the time of the study, this platform was selected due to the existing GeneXpert^®^ footprint in South Africa, through the Xpert^®^ MTB/RIF program which comprises 207 tuberculosis testing sites, and the goal of integrated diagnosis and monitoring through multipurpose testing platforms.

As part of the HIV VL testing mandate, technologies selected for the NHLS laboratories must be verified (“fit for purpose”) upon installation and prior to testing clinical specimens, regardless of placement within the testing framework. Verification material is frequently sought by the testing laboratory (laboratory networks) from residual patient’s specimens [39], but it is often difficult to obtain sufficient volumes for paired (duplicate/split) testing and is not always possible for remote testing sites. Participation in EQA programs, such as the global Virology Quality Assurance program (VQA, supplied by the Department of AIDS (National Institute of Health, Atlanta, GA, USA)) or the National External Quality Assessment Service (NEQAS, Sheffield, United Kingdom) HIV-1 RNA quantitation program, does provide assurance to an accredited laboratory for pathology services, but does not address pre-testing verification. Furthermore, these panels require expensive shipment, are only available at times of the annual panel testing cycles, and comprise limited numbers of specimens (n = ~5). In addition, the World Health Organisation has published considerations for POCT, including the need for instrument verification as ‘fit for purpose’ and external quality assessment at least annually [40]. Dried tube specimens (DTS) [41,42,43] were not selected, as it was desirable to minimize onsite processing, mimic plasma specimens as far as possible, and ensure sufficient specimen volume for use with the Xpert^®^ HIV-1 VL assay (1.1 mL).

In addition to the programs described above, the South African Viral Load Quality Assessment (SAVQA) panel [44] was previously developed to address the need for scaled HIV VL services in centralized HIV VL laboratories. This panel provides an accessible option for the verification of newly installed HIV VL testing platforms, initially the RealTime HIV-1 (Abbott Molecular, Des Plaines, IL, USA) and cobas^®^ AmpliPrep/cobas^®^ TaqMan^®^ (CAP/CTM; Roche Molecular, Pleasanton, CA, USA) assays, prior to testing clinical specimens, and has also been used for the rapid evaluation of new HIV VL assays [37,45,46,47]. The SAVQA panel [44] is a 42-specimen plasma panel prepared from purchased human plasma (known HIV-1 positive/negative) and quantified using RealTi*m*e HIV-1, CAP/CTM and cobas^®^ HIV-1 (Roche Molecular, Pleasanton, CA, USA). The panel is stored and shipped frozen, and only defrosted immediately prior to testing. The panel comprises 17 negative specimens and five repeats of five positive specimens with VL ranging from 2.7 log cp/mL to 5.0 log cp/mL. The panel was designed to measure accuracy, precision, carryover and limit of the blank [44]. The SAVQA panel was readily available, but was not suitable in its existing format. The panel required adaptation to avoid the need for cold-chain shipping and storage, with the remote testing sites having no refrigeration facilities. It was also desirable to include a smaller number of specimens to minimize cost and time constraints as the GeneXpert^®^ is a modular, cartridge-based system designed for random access, single specimen testing. We therefore designed a miniaturized, thermostable version of the SAVQA panel using a commercially available matrix, PrimeStore^®^ Molecular Transport Medium (MTM; Longhorn Vaccines and Diagnostics LLM, Bethesda, MD, USA), to allow ambient temperature shipping and storage. This medium achieved US FDA approval in 2018 [48], and has been evaluated with a variety of mycobacterial [49,50,51,52,53] and viral [54,55,56,57] specimens, including HIV [58]. In addition to the use of MTM-stored specimens with PrimeMix^®^ [50,55,56], MTM has been shown to be compatible with the Xpert^®^ MTB/RIF [52,56] and, more recently, the Xpert^®^ Xpress SARS-CoV-2 [57,59] assays (Cepheid, Sunnyvale, CA, USA), as well as the m2000 RealTime HIV-1 assay [58]. Verification panels were developed alongside a web-based result reporting tool, which was based on the web portal (www.tbgxmonitor.com) previously developed for Xpert^®^ MTB/RIF quality monitoring [60]. Following the successful verification rollout, an external quality assessment (EQA) panel was requested and was designed to measure pre- and post-processing analytics at these pilot laboratories. This manuscript aims to provide a detailed description of these pilot quality panels as an option for POCT HIV VL sites, using clinically relevant panel specimens which can be prepared centrally and sent to remote sites. These panels were specifically designed to meet the needs of remote testing laboratories using the Xpert^®^ HIV-1 VL assay, notably limited cold-chain shipping and cold-storage facilities on site, low throughput testing platforms, the need for ad hoc verification products and, frequently, lower-skilled laboratory staff. The use of QA materials, particularly when evaluated between laboratories, ensures that instruments are fit-for-purpose and that onsite processing is robust, thus ensuring best possible patient result quality within a tiered laboratory framework.

## 2. Materials and Methods

### 2.1. Panel Material Preparation

A SAVQA plasma panel, as described above, was removed from storage (−80 °C) and defrosted at ambient temperature, followed by brief centrifugation (3000 rpm, 1 min). HIV-negative specimens (1.3 mL) were not mixed with MTM to provide a clinically relevant specimen, overcoming the decreased viscosity/fat content of the MTM. The negative specimen is important to ensure that no cross-contamination occurs in either the reference laboratory or the testing laboratory during specimen preparation and testing. HIV-positive plasma specimens (300 µL) with known VL were added to 1 mL MTM (Longhorn Vaccines and Diagnostics LLC, Bethesda, MD, USA), giving a dilution factor of 4.3 (total volume/specimen volume). To minimize the risk of leakage, each specimen was packaged individually in a sealed plastic bag with an absorbent pad and the complete panel was then placed into a second sealable bag. Specimens were shipped at ambient temperature using the routine NHLS specimen transport system.

**Figure 1:**
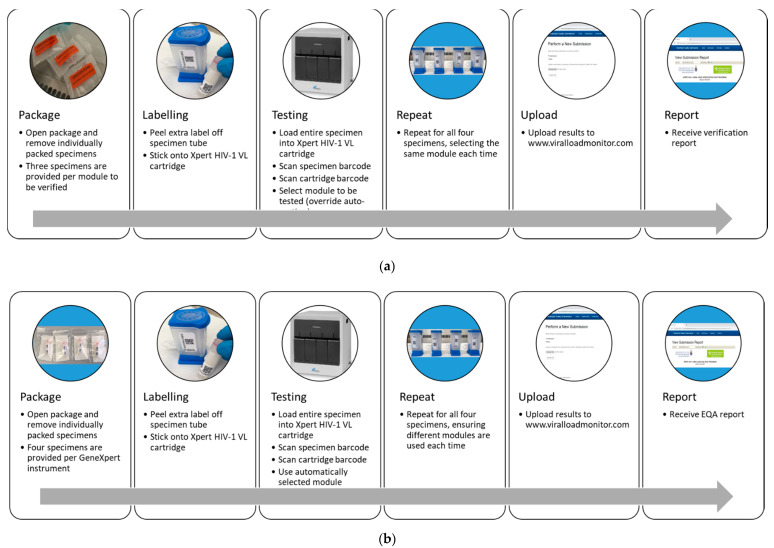
Processing of Verification and EQA panels. (**a**) Verification panel: same module must be used for each set of specimens. Verification panels are labelled with orange labels to remind users of this. (**b**) EQA panel: different modules must be used for each specimen. HIV: human immunodeficiency virus. VL: viral load. cp/mL: copies per milliliter. EQA: external quality assessment.

Two panel formats were designed: (i) a verification panel (Figure 1a) and (ii) an EQA panel, (Figure 1b). The verification panel was used to ensure that instruments were functioning correctly upon installation, instrument (module) replacement or instrument movement, and can also be used for staff training. The verification panel consisted of three specimens per module tested: two specimens of known HIV VL stabilized in MTM buffer and one HIV-negative specimen (plasma only). The target ranges for the HIV-positive specimens were 2.7 log cp/mL (low), 3.0 log cp/mL (low), 4.7 log cp/mL (high), and 5.0 log cp/mL (high). All sites received one low VL specimen, one high VL specimen and one HIV-negative specimen, as per testing organization requirements. The EQA panel was necessary for ongoing monitoring of instruments and testing sites. Four specimens were provided per instrument tested, with an instrument being defined as “up to four” GeneXpert^®^ systems attached to one computer. The panel included three specimens of a known HIV VL stabilized in MTM buffer, with a target range of 3.0 log cp/mL, 3.7 log cp/mL and 4.7 log cp/mL, and one HIV-negative plasma specimen. On preparation of either panel format, one specimen in each range was tested using the reference laboratory GeneXpert^®^ instrument (reference specimen; day 0).

### 2.2. Xpert^®^ HIV-1 VL Quality Panel Testing

Both the verification and EQA specimens were processed according to the Xpert^®^ HIV-1 VL manufacturer’s instructions (Cepheid, Sunnyvale, CA, USA), using the liquid panel in place of clinical plasma. Briefly, the Xpert^®^ HIV-1 VL cartridge was opened and the entire specimen volume (1.3 mL) was transferred into the Xpert^®^ HIV-1 VL cartridge using a precision pipette or 1 mL Pasteur pipette (supplied by Cepheid as part of the kit). The specimen barcode and cartridge number were scanned, and the specimen was tested using the Xpert^®^ HIV-1 Viral Load assay definition file. The original SOP did not include centrifugation instructions, but this was amended after the first verification panel was analysed to ensure that every specimen was briefly centrifuged (3000 rpm, 1 min) prior to processing.

### 2.3. Result Return and Performance Scoring

A web portal (www.viralloadmonitor.com), based on the original TBGxMonitor website [60] for upload of both verification and EQA results and report generation, was created in collaboration with SmartSpot Quality Pty (Ltd.) (Johannesburg, Gauteng, South Africa). Users were required to upload the comma-separated values (CSV) run files (automatically produced by the GeneXpert^®^ software) for the Xpert^®^ HIV-1 VL panel specimens using a USB device. Results were converted using the dilution factor (4.3) and this was applied within the website logic as part of the scoring algorithm. The criteria for designing the panels were based on monitoring across the clinically relevant threshold of 1000 cp/mL [5], and therefore the scoring system and performance monitoring were applied to this critical range. This included evaluating acceptable differences between the test specimen and the Xpert^®^ HIV-1 VL reference specimen (described above), and was originally defined as <1.0 log cp/mL difference. This large variability was selected to account for potential artefacts generated by specimen dilution, ambient temperature shipping and result conversion. Retrospective analyses at <0.5 log cp/mL difference and <0.3 log cp/mL difference, in line with generally accepted VL variation [61,62], were also performed. Finally, the Xpert^®^ HIV-1 VL reference VL was compared to the pooled mean VL achieved by the 13 testing sites, ensuring that the reference laboratory instrument was performing acceptably and that the reference result was suitable for use as the standard. The scoring system was aligned with the previously well-described TB quality program [60,63,64] and, although differences exist between qualitative (TB) and quantitative (VL) result outputs, the performance was similarly applied due to the modular nature of the GeneXpert system, as follows: each specimen tested received a score out of two: correct result (2/2); error, invalid, >1.0 log cp/mL quantifiable result bias (1/2); incorrect result (e.g., HIV positive reported as HIV negative: 0/2). Each panel was then scored out of six for verification and out of eight for EQA. Scoring logic is detailed in Table 1. The overall panel performance across all sites was measured by the mean, median, range and standard deviation (SD) of the quantifiable viral loads, which were calculated using Microsoft^®^ Excel^®^ 2016 (Microsoft Corporation, Redmond, WA, USA). Regression, the concordance correlation coefficient (ρ_c_) [65,66], including a Pearson correlation coefficient (*p*; measure of precision) and a bias correction factor (C_b_; measure of accuracy), and Bland–Altman [67,68] analyses were performed and graphically represented using MedCalc Statistical Software version 18.11 (MedCalc Software bvba, Ostend, Belgium; http://www.medcalc.org; 2018).

### 2.4. Verification and EQA Pilot Field Evaluation

The pilot evaluation was nested within a field trial of near-patient VL testing, overseen by the NHLS NPP (Johannesburg, South Africa). Thirteen district laboratory facilities were selected and provided with a GeneXpert^®^ IV (Cepheid, Sunnyvale, CA, USA). The laboratories were located in remote areas across six provinces (Eastern Cape: *n* = 2, Northern Cape: *n* = 4, Western Cape: *n* = 3; Free State: *n* = 1, Limpopo: *n* = 2; North West Province: *n* = 1). Technicians were recruited and received training on the GeneXpert^®^ platform and the Xpert^®^ HIV-1 VL assay. The verification and EQA material were designed to meet requirements of the NPP to ensure that the instruments were fit-for-purpose and that specimen processing was being correctly performed.

Verification panels (*n* = 4 per site) were provided to all sites in September 2017, following instrument installation and prior to patient testing. Further verification panels (*n* = 5) were provided on an ad hoc basis as modules were replaced. EQA panels (*n* = 1 per site) were provided to the sites in June and November 2018. For the pilot evaluation, the automatically generated reports were manually checked prior to release, but the website has the capacity to automatically release reports to the sites.

### 2.5. Stability Testing

Prior to initial supply to sites, verification specimens (2.7 log cp/mL; 5.0 log cp/mL) were prepared and tested in duplicate at days 7, 14, 21, and 28 (as per process described above) to determine stability compared to the day 0 reference result. Extra EQA panels (3.0 log cp/mL, 3.7 log cp/mL and 4.7 log cp/mL) were prepared at the same time as those sent to the sites and tested at days 24, 43, 84 and 150 post manufacture to determine longer term stability. All specimens were stored at ambient temperature in sealed plastic bags with desiccant.

## 3. Results

### 3.1. Verification Panel Performance

**Figure 2:**
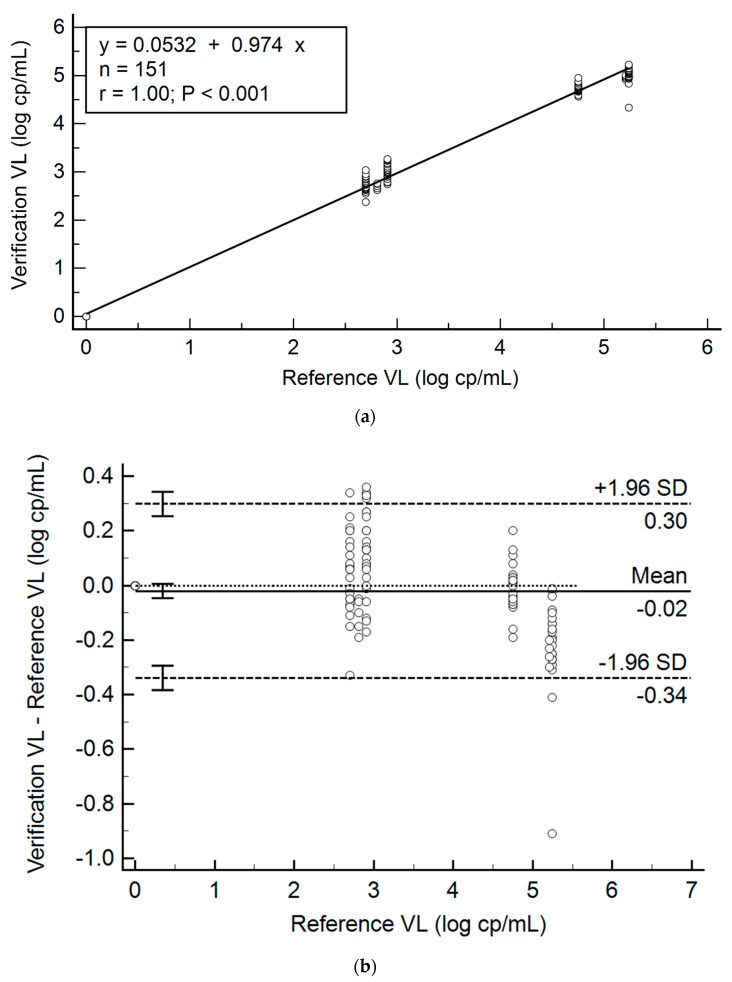
Verification panel VL variation (log cp/mL) across different testing sites (*n* = 13). (**a**) Regression analysis for all verification panels tested between September 2017 and November 2018. (**b**) Bland-Altman agreement of the viral load results, compared to the reference result obtained at panel preparation. One outlier specimen (4.33 log cp/mL; −0.91 log cp/mL difference from reference VL) was noted in the 5.0 log cp/mL category, but was within the acceptable range for the pilot panels (<1.0 log cp/mL).VL: viral load. cp/mL: copies per milliliter. SD: standard deviation.−

All sites tested and uploaded results to the website within three days of panel receipt. Result scores and outcomes are summarized in Table 2 and Figure 2, with detailed information provided in Supplementary Table S1. Quantifiable VL results were within acceptable limits for verification (<1.0 log cp/mL difference from the reference VL, as shown in Table 2) and all reference results were within 0.3 log cp/mL of the pooled mean VL of the specimens tested, although it was noted that the VL bias was high in the 5.0 log cp/mL reference specimen (0.22 log cp/mL). In addition, the sites’ verification VL results were compared to the mean VL (data not shown) and this was comparable to analysis using the reference VL values. The ρ_c_ across all sites (n = 151 specimens) was 0.997 (95% confidence interval [CI]: 0.995 to 0.998), with a *p* of 0.997 and a C_b_ of 0.999. The mean bias was −0.02 log cp/mL (95% CI: −0.046 to 0.006), with a coefficient of determination (R^2^) value of 0.9940.

The error rate (20/171; 11.7%) for the verification panels was higher than expected, and was primarily a result of processing errors (55% of errors). Seven errors (35%) were linked to the internal probe failures, two to syringe pressure (10%) and eleven relating to input volume (errors 2096 (35%) and 2097 (20%)). The majority of errors reported (13/20; 65%) occurred in the clinically relevant negative specimen, indicating laboratory processing errors. It was determined, on discussion with the program manager, that the specimens were not being centrifuged prior to testing and that incorrect pipetting procedures may have contributed to the errors. Changes were made to the standard operating procedure (i.e., to centrifuge all specimens prior to use, as would be required for clinical specimens) and staff retraining was performed if necessary. Once these changes were implemented, the error rate (over ad hoc verification and EQA) decreased to 1.7% (2/119 further tests), indicating that correct operating procedures were being observed.

### 3.2. Pilot EQA Performance

**Figure 3:**
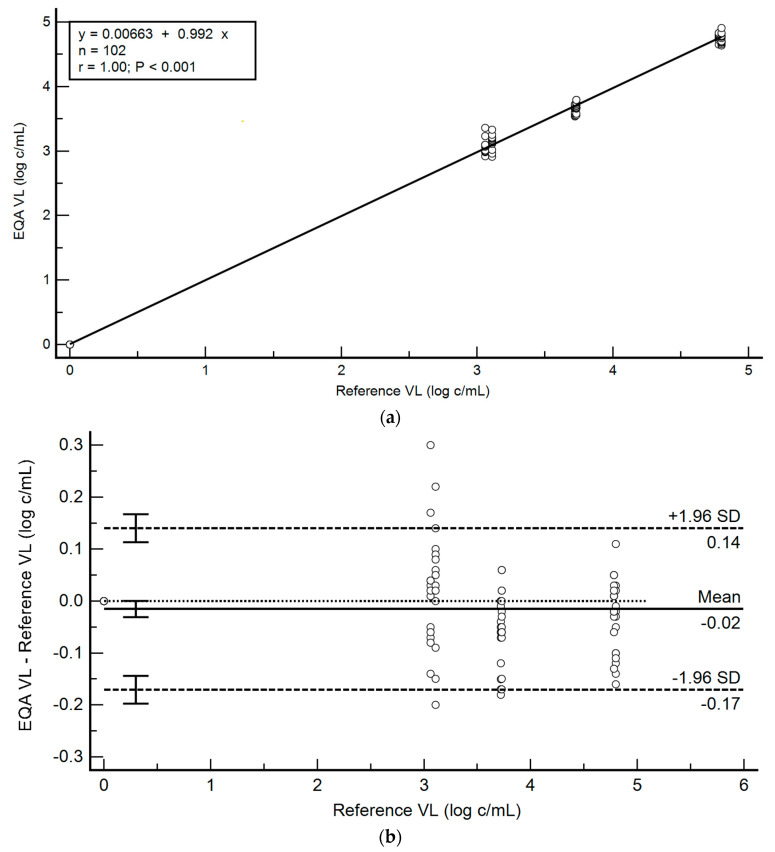
EQA Panel VL variation (log cp/mL) across different testing sites (n = 13) and EQA panels (*n* = 2). (**a**) Regression analysis for EQA Panels 1 and 2 (*n* = 102/104 specimens). (**b**) Bland-Altman agreement of the viral load results (*n* = 102/104 specimens), compared to the reference result obtained at panel preparation. EQA: external quality assessment. VL: viral load. cp/mL: copies per milliliter. SD: standard deviation.

Two cycles of EQA (E18V1, E18V2) were shipped to 13 sites (18 June 2018, 12 November 2018) and results were uploaded within seven days (mean: 4.1 days). All sites showed acceptable performance across both EQA panels; the program performance is summarized in Table 3 and Figure 3, and complete site results are detailed in Supplementary Table S2. Viral loads were within acceptable limits for EQA (<1.0 log cp/mL bias), and all negative specimens were reported as not detectable (no carryover). The ρ_c_ for the EQA pilot panels (two EQA panels, n = 102/104 specimens) across all sites was 0.9985 (95% CI: 0.9978 to 0.9990), with a ρ_c_ of 0.9987 and a C_b_ of 0.9998. The mean bias was 0.03 (95% CI: 0.02 to 0.05). The error rate was 1.9% (two in 104 tests) and was caused by volume loading (user) errors.

### 3.3. Retrospective Result Analysis

Retrospective analysis of the verification and EQA results was performed after the pilot evaluation, in order to accommodate acceptable VL biases [61,62]. Amongst 107 quantifiable verification results, ten (9.3%) showed a bias of >0.3 log cp/mL (range: 0.36, −0.91). Only one outlier specimen (4.33 log cp/mL) displayed a bias >0.5 log cp/mL: −0.91 log cp/mL compared to the reference VL and −0.69 log cp/mL compared to the pooled mean VL. This specimen was part of the 5.0 log cp/mL group, where the reference VL (5.24 log cp/mL) was notably higher than the pooled mean VL (5.02 log cp/mL). A second outlier (4.83 log cp/mL) in this group had a VL bias of −0.41 log cp/mL compared to the reference VL, with an acceptable bias of −0.19 log cp/mL compared to the pooled mean VL. Only three specimens (2.8%) had a bias of >0.3 log cp/mL compared to the pooled mean VL. All quantifiable EQA VL (n = 76) results showed a bias of <0.3 log cp/mL compared to the reference VL.

### 3.4. Specimen Stability

**Figure 4:**
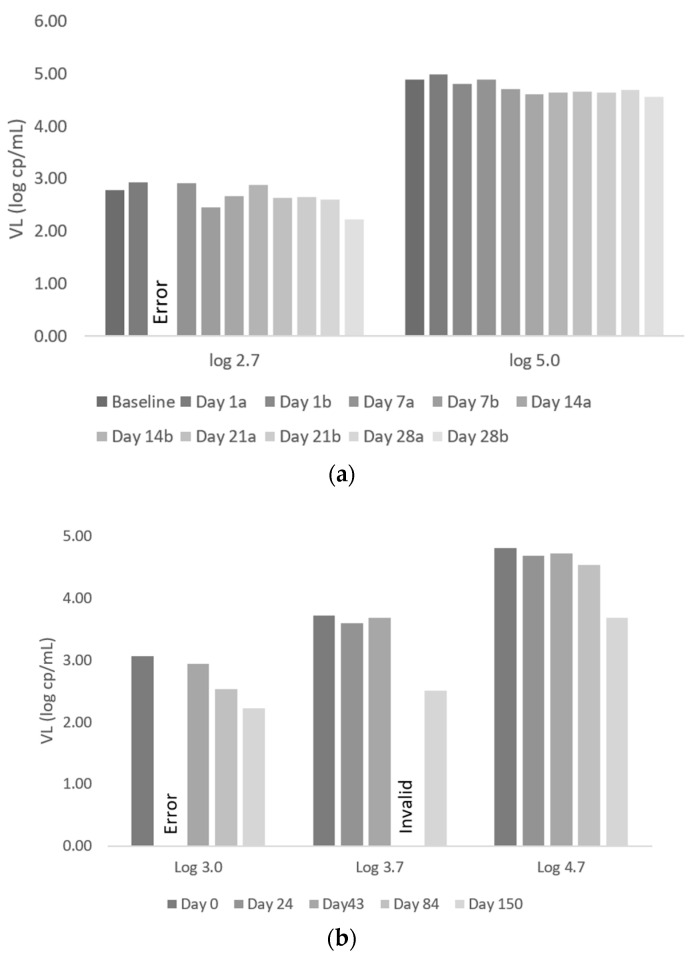
Stability of EQA Pilot Panel Baseline to Day 150. (**a**) Bar chart showing VL from Day 0 to Day 28, with specimens tested in duplicate. There is little VL variability. (**b**) Bar chart showing VL from Day 0 to Day 150. There is a decrease in VL between Day 84 and Day 150. The VL remains within 0.2 log cp/mL of the expected VL for the log 3.7 and log 4.7 specimens until Day 84. There is a decrease at Day 84 for the log 3 specimen, but it remains within 0.5 log cp/mL of the expected VL. By Day 150, all VL exceed >0.5 log cp/mL difference from day 0, with both the log 3.7 and log 4.7 specimens showing a VL decrease of >1.0 log cp/mL. EQA: external quality assessment. VL: viral load. cp/mL: copies per milliliter.

Stability of the specimens stored in MTM was evaluated prior to panel design and supply, with specimen stability acceptable up to 28 days (Figure 4a). Testing of EQA panels in the reference laboratory between weeks 4 and 20 (Figure 4b), showed stability of all specimens at week 6 (day 43) and extended stability of the higher VL range (4.7 log cp/mL) specimens until week 12 (day 84). However, by week 12, a decrease of ~0.5 log cp/mL was noted in the lower (3.0 log cp/mL) VL range. Errors were noted in the 2.7 log cp/mL on day 1 (repeat) and the 3.0 log cp/mL specimen at day 24 (both error 2126; module reset), and in the 3.7 log cp/mL specimen at day 84 (invalid, error 5016: probe check error). These relate to the instrument and the cartridge, rather than the specimen. Retesting was not possible due to limited specimen availability. By Day 150, all VL exceeded >0.5 log cp/mL difference from baseline (day 0), with both the 3.7 log cp/mL and 4.7 log cp/mL specimens showing a VL decrease of >1.0 log cp/mL. Bland-Altman analysis of the reportable VL results (*n* = 14/16) over the weeks, including day 84, when a VL decrease was noted, but excluding day 150, when VL were no longer relevant, gave a mean bias of −0.06 log cp/mL with a lower limit of −0.34 log cp/mL (95% CI: −0.89 to −0.21) and an upper limit of 0.23 log cp/mL (95% CI: 0.10 to 0.77). Including day 150 (*n* = 18/20) gave a mean bias of −0.20 log cp/mL with a lower limit of −0.97 log cp/mL (95%CI: −2.11 to −0.62) and an upper limit of 0.58 log c/mL (95% CI: 0.23 to 1.72), beyond acceptable limits for supply to sites.

## 4. Discussion

Laboratory quality monitoring is vital to ensure ongoing patient result testing accuracy [39,69]. Instruments must be evaluated prior to implementation, verified before use in the field and monitored on an ongoing basis. Similarly, staff competency should be evaluated through training, observation and participation in quality programs. Evaluation can be performed on existing specimens (e.g., frozen plasma), prospective specimens (against a reference instrument currently in use) or on well-described quality panels (e.g., NEQAS, SAVQA). EQA, through supply of standardized specimens for testing and through continuous quality monitoring (CQM, e.g., analysis of central data repositories), enables program managers to identify potential instrument or staff deficiencies for correction. Participation in EQA programs has been shown to improve participant performance [42]. CQM of assays and instruments is becoming standard practice for many connected diagnostics. Operational dashboards, such as C360 (Cepheid), provide assay and instrument quality information on errors, utility, and various result parameters on a module/instrument/laboratory and location basis, and can be utilized for daily and monthly monitoring to identify quality issues, without waiting for EQA panel cycles [70]. CQM, through the C360 platform, was successfully applied during the near-patient testing pilot into which this evaluation was nested, but is beyond the scope of this manuscript. EQA is complimentary to CQM, ensuring ongoing pre- and post-analytical performance monitoring, which is particularly important where staff turn-over is high.

The Xpert^®^ HIV-1 VL assay was previously evaluated, using both the SAVQA panel and clinical specimens [37], and has since been extensively evaluated in the field [14,71], meaning that the assay did not require further evaluation prior to implementation. However, before the implementation pilot could commence, verification of the modules was required, and was complicated by the remote placement of the instruments as residual plasma specimens were not readily available. Alternative options for instrument verification were thus needed. This manuscript describes the design and pilot evaluation of quality panels used for POCT HIV VL. The panels were designed to meet specific requirements: (i) specimen processing needed to be as similar as possible to actual specimens; (ii) thermostable transport and storage; (iii) reproducible VL results, such that processing or instrument issues could be detected during verification and ongoing EQA, and (iv) safe during transport. While initially designed for module verification, the panels were easily adapted for ongoing EQA. These panels were based on similar principles to the Xpert^®^ MTB/RIF program [63,64], which has been used successfully throughout the NHLS to monitor 207 Xpert^®^ MTB/RIF testing sites, as well as internationally (28 countries), and was expected to provide similar rigorous quality monitoring to Xpert^®^ HIV-1 VL sites.

It is notable that the panels were supplied in a liquid format and that no processing was required beyond centrifugation and direct addition of specimen into the Xpert^®^ HIV-1 cartridge, mimicking routine patient specimen testing. This was in contrast to dried tube specimens (DTS), which have been used throughout sub-Saharan Africa for EQA [41,42,43]. DTS were not selected for this pilot as the NPP preferred to minimize specimen processing variability during specimen reconstitution by using a liquid panel, although DTS met all other requirements described. Furthermore, similarly to the original SAVQA panel, the verification program was designed for rapid deployment using local resources, decreasing reliance on scheduled schemes [44]. Shipping of liquid specimens is potentially problematic, given the risk of leakage, particularly if the transport infrastructure is poor (e.g., degraded road surfaces). Panels were well packaged and no leakage of the specimen from the tube into the protective packing was observed. However, the extra packaging, as described above, is recommended for similar panels going forward to minimize risk to transport personnel and to meet IATA requirements [72]. The infectivity of HIV when stored in MTM was not tested in this pilot, but existing studies have shown that pathogens are fully inactivated on addition to the buffer [48,50,54,57,73], while RNA integrity is simultaneously preserved [50,55,56,57], including HIV-1 RNA [58].

Thermostability of the panels, with little VL variation, was shown for a minimum of twelve weeks from manufacture. Earlier studies have shown that viral RNA (e.g., influenza) can be reliably detected for up to 196 days [54] and quantified for up to 23 days [56]. This study has shown longer-term stability on HIV RNA, although it should be noted that the manufacturer only recommends storage at ambient temperature for 30 days. Furthermore, stability testing was performed in Johannesburg during the South African winter and spring, with temperatures ranging from 8 to 23 °C, but with minimal humidity. More recent studies performed during the hotter months (maximum temperature 31 °C) and with increased humidity showed decreases of >1 log cp/mL by 10 weeks (personal communication, Dean Sher, SmartSpot Quality Pty (Ltd.), Johannesburg, Gauteng, South Africa) and is therefore a consideration for long-term stability in warmer climates. A recent manuscript reported decreased yield of Mycobacterium tuberculosis in oral mucosa specimens stored in PrimeStore MTM after 30 days and also after extended freezing [52], a finding that may similarly affect these specimens if frozen. Further stability evaluations in humid and warmer settings are recommended to ensure similar stability in such settings. In this evaluation, the QA specimens were made to order and generally tested within one week. It should also be noted that the dilution factor was applied to this pilot in order to allow for a comparison with the original SAVQA data.

In order to determine if specimen variability [74] affected the performance of sites compared to the reference VL, the specimen VL from all sites and the reference VL were compared to the pooled mean VL of all sites. In all cases, the mean VL and the reference VL were similar (−0.02 log cp/mL mean difference), although the reference instrument did produce a higher VL (5.24 log cp/mL vs 5.02 log cp/mL) than all sites in the 5.0 log cp/mL range. This was not clinically significant and did not affect site performance outcome. The bias of the single outlier specimen described (−0.91 log cp/mL bias) was acceptable for verification in terms of the panel design, but unacceptable in the retrospective analysis. However, the site still achieved a module score of 5/6 in the retrospective analysis and patient specimen testing could commence. The benefit of a quality program across multiple sites was that multiple instruments were tested concurrently and panels could be compared to the pooled mean VL rather than only the reference VL; this provided an additional quality control of the reference instrument and the potential to highlight unexpected instability of the quality material. Retrospective analysis of the specimens showed that they could be evaluated at 0.3 and 0.5 log cp/mL bias [61,62], and these thresholds should be implemented when using this quality panel further.

This design can be adapted to tiered laboratory systems to ensure continued quality POCT HIV VL testing, although resources (MTM buffer, plasma (if purchased), staff time required to manufacture the panels and to collate the results, post-manufacture quality testing and shipping) and individual country needs must be evaluated on an individual basis [69]. Similarly, if this quality material was adapted by commercial suppliers, the cost and feasibility of scaled manufacture at an implementation price acceptable to countries needing such QA products should be investigated. Of note, is the limited stability and compatibility with alternative HIV VL assays if using assays beyond Xpert^®^ HIV-1 VL. This was not evaluated during this pilot, but it has been observed that the MTM buffer occasionally interacts negatively with certain HIV VL assays (personal communication, Dean Sher, SmartSpot Quality Pty (Ltd.), Johannesburg, Gauteng, South Africa). The value of formal verification or EQA panels should not be disregarded, particularly for smaller programs where globally standardized specimens may provide more rigorous quality measures [39,69], but mandatory participation in such schemes varies [39]. A further consideration for using commercial EQA panels is to free up the time of the program managers from producing panels and evaluating results, so as to use this time to assist the laboratories which the EQA identifies as needing help, to identify root-causes and implement corrective actions [69]. Ultimately, whether in-house or commercial, the goal is to ensure quality laboratory testing [39,69], which impacts positively on patient care and management.

Ongoing quality monitoring at all levels of a tiered laboratory network is paramount to ensure that patient results are accurate. This can be difficult for POCT instruments placed in remote settings, where quality management options used in centralized laboratories are not feasible, but where quality monitoring is vital. The quality panels described in this manuscript provide simple and convenient verification and/or EQA options for countries aiming to implement Xpert^®^ HIV-1 VL.

## Figures and Tables

**Table 1:** Summary of scoring logic.

Specimen Score	Results	Outcome
2/2	Correct result	Pass
1/2	Error, Invalid, No result >1.0 log cp/mL quantifiable result bias	Acceptable
0/2	Incorrect result (e.g., HIV positive reported as HIV negative)	Concern
**Verification Score**	**Percentage Performance**	**Outcome**
6/6	100%	Pass
5/6	83.3%	Acceptable
≤4/6	66.7%	Unacceptable
**EQA Score**	**Percentage Performance**	**Outcome**
8/8	100%	Pass
7/8	87.5%	Acceptable
6/8	75.0%	Concern
≤5/8	62.5%	Unacceptable

The table is divided into specimen score, verification score and EQA score sections (shown in bold). Specimen Score: Each specimen generates a score out of two. Verification Score: Verification of a module generates a score out of six (three specimens per module). EQA Score: EQA of an instrument generates a score out of eight (four specimens per instrument, run over different modules). If an unacceptable score is obtained, the site is required to conduct a root cause analysis and corrective action, and to test a second verification or EQA panel. Site trainers or monitors may provide further interventions (e.g., staff training, instrument calibration). EQA: external quality assessment. HIV: human immunodeficiency virus. cp/mL: copies per milliliter.

**Table 2:** Site Verification Summary: September 2017–November 2018 (compared to reference VL).

Panel	Expected Viral Load (log cp/mL)	Reference Viral Load (log cp/mL)	Tested (n)	Result Obtained (n (%))	Viral Load Bias (Mean (Median) Range) (log cp/mL)	Standard DeViation of Mean Bias (log cp/mL)	Error (n)	Invalid (n)	Reference vs. Mean (log cp/mL)
**1**	Negative	Negative	52	39 (75.0)	0	0	12	1	0
**2 ^c^**	Negative	Negative	5	5 (100)	0	0	0	0	0
**1**	2.70	2.70	26	23 (88.5)	0.04 (0.06) −0.33, 0.34	0.15	2	1	−0.04
**2 ^c^**	2.70	2.81	5	5 (100)	−0.11 (−0.10)) −0.19, −0.06	0.06	0	0	0.11
**Overall (log 2.70)**		-	31	28 (90.3)	0.02 (−0.02) −0.33, 0.34 ^b^	0.15	2	1	-
**1**	3.00	2.91	26	25 (96.2)	0.13 (0.13) (−0.17, 0.36)	0.16	1	0	−0.14
**1**	4.70	4.75	26	25 (96.2)	−0.01 (0.00) −0.19, 0.20	0.09	1	0	0.01
**1**	5.00	5.24	26	25 (96.2)	−0.22 (−0.20) −0.91 ^a^, −0.01	0.18	1	0	0.22 ^a^
**2 ^c^**	5.00	5.21	5	4 (80.0)	−0.25 (−0.25) −0.30; −0.20	0.04	0	1	0.25
**Overall (log 5.00)**		-	31	29 (93.6)	−0.23 (−0.22) −0.91; −0.01	0.17	1	1	-
**Overall (57 verification panels)**			171	151/171 (88.3) Quantified: 107/114 (93.9)	−0.02 (0.00) (−0.91, 0.36)	0.16	17 9.9%	3 1.8%	0.07

^a^ increased variability owing to one outlier specimen (4.33 log cp/mL). If this specimen is excluded, the mean bias increases to −0.19 log cp/mL with a range of −0.41 to −0.01, and the difference between the reference and the pooled mean decreases to 0.19 log cp/mL. ^b^ variation around the median >0.30 when two panels are combined, but remains <0.03 log cp/mL in the individual panels. ^c^ verification panel 2 numbers are low (n = 5), so values lack robustness, but are similar to the larger panel 1. n: number. cp/mL: copies per milliliter.

**Table 3:** Site EQA Summary: September 2017–November 2018.

Panel	Expected Viral Load (log cp/mL)	Reference Viral Load (log cp/mL)	Tested (n)	Result Obtained(n (%))	Viral Load Bias (Mean (Median) Range) (log cp/mL)	Standard Deviation of Mean Bias(log cp/mL)	Error (n)	Reference vs. Mean(log cp/mL)
**1**	Negative	Negative	13	13 (100)	0	0	-	0
**2**	Negative	Negative	13	13 (100)	0	0	-	0
**1**	3.00	3.06	13	12 (92.3)	0.02 (0.02) −0.14, 0.30	0.11	1	−0.02
**2**	3.00	3.11	13	13 (100)	0.02 (0.04) −0.20, 0.22	0.12	-	0.05
**Overall (log 3.00)**		3.09	26	25 (96.2)	0.02 (0.02) −0.20, 0.30	0.11	1	-
**1**	3.70	3.72	13	13 (100)	−0.06 (−0.06) −0.18, 0.05	0.07	-	0.06
**2**	3.70	3.73	13	13 (100)	−0.04 (−0.04) −0.17, 0.06	0.07	-	0.01
**Overall (log 3.70)**		3.73	26	26 (100)	−0.05 (−0.05) −0.18, 0.06	0.07	-	-
**1**	4.70	4.80	13	13 (100)	−0.05 (−0.04) −0.16; 0.11	0.08	-	0.05
**2**	4.70	4.78	13	12 (92.3)	−0.01 (0.01) −0.13, 0.05	0.05	1	−0.02
**Overall (log 4.70)**		4.79	26	25 (96.2)	−0.03 (−0.02) −0.16; 0.11	0.07	1	-
**Overall (26 EQA panels panels)**			104	102/104 (98.1)	−0.02 (−0.02) −0.20, 0.30	0.09	2 1.9%	-

EQA: External quality assessment. n: number. cp/mL: copies per milliliter.

## Supplementary Materials

The following are available online at https://www.mdpi.com/2075-4418/11/2/160/s1, Supplementary Table S1: Detailed Site Verification Summary: September 2017–November 2018; Supplementary Table S2: Detailed Site EQA Summary: September 2017–November 2018.
