# Supplementary material for: The Development of a Standardized Quality Assessment Material to Support Xpert® HIV-1 Viral Load Testing for ART Monitoring in South Africa
Source: Diagnostics (Basel). 2021 Jan 22;11(2):160. doi: 10.3390/diagnostics11020160 (PMC7911816; doi:10.3390/diagnostics11020160)
Supplement: Supplementary file 1 [file diagnostics-11-00160-s001.pdf]

Supplementary Table S1: Detailed Site Verification Summary: September 2017-November 2018

Modules verified at each of thirteen sites are shown, with error codes highlighted. The initial verification panels had a high number of volume errors, which led to operating procedure changes. A score of ≤4/6 was unacceptable and the module required retesting. Replaced modules were also tested before patient specimen testing commenced.

Site/Panel	Module	Expected HIV VL	Difference (log cp/mL)	Score	Outcome
**Site 1**	A1	Negative	0.00	6/6	Pass
		Low	0.11		
		High	0.29		
	A2	Negative	Error2097	5/6	Acceptable
		Low	-0.10		
		High	0.08		
	A3	Negative	0.00	6/6	Pass
		Low	-0.20		
		High	0.19		
	A4	Negative	0.00	6/6	Pass
		Low	-0.20		
		High	0.31		
**Site 2**	A1	Negative	0.00	6/6	Pass
		Low	-0.11		
		High	0.09		
	A2	Negative	0.00	6/6	Pass
		Low	-0.08		
		High	-0.04		
	A3	Negative	0.00	6/6	Pass
		Low	0.05		
		High	0.06		
	A4	Negative	0.00	6/6	Pass
		Low	-0.20		
		High	0.91		
**Module Replacement**	A3	Negative	0.00	5/6	Acceptable
		Low	-0.05		
		High	Invalid		
**Site 3 V17V11WA1**	A1	Negative	0.00	6/6	Pass
		Low	-0.07		
		High	0.27		
	A2	Negative	0.00	6/6	Pass
		Low	0.12		
		High	-0.03		
	A3	Negative	0.00	6/6	Pass
		Low	-0.21		
		High	-0.01		

	A4	Negative	Error2097	5/6	Acceptable
		Low	-0.27		
		High	0.02		
**Module Replacement**	B3	Negative	0.00	6/6	Pass
		Low	-0.10		
		High	-0.26		
**Site 4**	A1	Negative	0.00	6/6	Pass
		Low	0.33		
		High	0.25		
	A2	Negative	0.00	6/6	Pass
		Low	-0.27		
		High	0.05		
	A3	Negative	0.00	5/6	Acceptable
		Low	0.15		
		High	Error2005		
	A4	Negative	0.00	6/6	Pass
		Low	-0.16		
		High	0.14		
**Site 5**	A1	Negative	0.00	5/6	Acceptable
		Low	Error5017		
		High	0.19		
	A2	Negative	0.00	6/6	Pass
		Low	0.01		
		High	-0.11		
	A3	Negative	0.00	6/6	Pass
		Low	-0.22		
		High	-0.01		
	A4	Negative	0.00	6/6	Pass
		Low	-0.15		
		High	0.27		
**Site 6**	A1	Negative	0.00	6/6	Pass
		Low	0.01		
		High	0.20		
	A2	Negative	Error2096	5/6	Acceptable
		Low	-0.34		
		High	0.07		
	A3	Negative	Error2096	5/6	Acceptable
		Low	0.05		
		High	-0.13		
	A4	Negative	Error2096	5/6	Acceptable
		Low	-0.13		
		High	0.14		
**Site 7**	A1	Negative	0.00	5/6	Acceptable
		Low	Error2005		
		High	0.04		
	A2	Negative	0.00	6/6	Pass
		Low	-0.03		
		High	-0.01		

	A3	Negative	0.00	6/6	Pass
		Low	-0.03		
		High	-0.08		
	A4	Negative	0.00	5/6	Acceptable
		Low	Error5007		
		High	0.25		
**Module Replacement**	A4	Negative	0.00	6/6	Pass
		Low	-0.15		
		High	-0.19		
**Site 8**	A1	Negative	0.00	6/6	Pass
		Low	-0.20		
		High	0.01		
	A2	Negative	0.00	6/6	Pass
		Low	0.06		
		High	0.04		
	A3	Negative	0.00	6/6	Pass
		Low	0.07		
		High	0.16		
	A4	Negative	0.00	6/6	Pass
		Low	-0.07		
		High	0.17		
**Site 9**	A1	Negative	Error2097	5/6	Acceptable
		Low	-0.25		
		High	0.41		
	A2	Negative	Error5017	5/6	Acceptable
		Low	-0.13		
		High	0.05		
	A3	Negative	0.00	6/6	Pass
		Low	-0.06		
		High	0.07		
	A4	Negative	Invalid (5016)	5/6	Acceptable
		Low	-0.34		
		High	0.27		
**Site 10**	A1	Negative	0.00	6/6	Pass
		Low	-0.16		
		High	0.12		
	A2	Negative	0.00	6/6	Pass
		Low	-0.32		
		High	-0.03		
	A3	Negative	0.00	6/6	Pass
		Low	0.03		
		High	0.00		
	A4	Negative	Error2096	4/6	Unacceptable
		Low	-0.1		
		High	Error5017		

**Verification Repeat**	A4	Negative	0.0	6/6	Pass
		Low	0.1		
		High	0.1		
**Site 11**	A1	Negative	Error2096	5/6	Acceptable
		Low	-0.06		
		High	0.25		
	A2	Negative	0.00	6/6	Pass
		Low	0.13		
		High	0.03		
	A3	Negative	0.00	6/6	Pass
		Low	0.05		
		High	-0.02		
	A4	Negative	Error2096	5/6	Acceptable
		Low	0.17		
		High	0.20		
**Site 12**	A1	Negative	0.00	5/6	Acceptable
		Low	Invalid (5016)		
		High	0.27		
	A2	Negative	0.00	6/6	Pass
		Low	-0.36		
		High	0.06		
	A3	Negative	0.00	6/6	Pass
		Low	-0.14		
		High	-0.20		
	A4	Negative	0.00	6/6	Pass
		Low	-0.25		
		High	0.10		
**Module Replacement**	A4	Negative	0.00	6/6	Pass
		Low	-0.19		
		High	-0.30		
**Site 13**	A1	Negative	0.00	6/6	Pass
		Low	-0.34		
		High	0.16		
	A2	Negative	Error2097	5/6	Acceptable
		Low	-0.33		
		High	0.05		
	A3	Negative	0.00	6/6	Pass
		Low	0.08		
		High	-0.02		
	A4	Negative	Error2096	5/6	Acceptable
		Low	0.00		
		High	0.22		
**Module Replacement**	A1	Negative	0.00	6/6	Pass
		Low	-0.06		
		High	-0.23		

Supplementary Table S2: Detailed Site EQA Summary: September 2017-November 2018

EQA results from instruments tested at each of thirteen sites are shown, with error codes highlighted. A score of ≤7/8 was unacceptable and it was recommended that EQA was repeated after root cause analysis and corrective actions.

Site	Score	Outcome	Comment	Log Difference (copies/ml)			
				Neg	Log 3.0	Log 3.7	Log 4.7
**EQA Round 1 2018**							
**1**	8/8	Pass		0	-0.30	0.00	0.05
**2**	8/8	Pass		0	-0.03	0.12	0.10
**3**	8/8	Pass		0	0.14	0.15	0.01
**4**	8/8	Pass		0	-0.04	0.04	0.16
**5**	8/8	Pass		0	-0.01	0.06	0.02
**6**	7/8	Acceptable	Load volume error	0	ERR-2097	0.07	-0.03
**7**	8/8	Pass		0	0.05	0.00	0.10
**8**	8/8	Pass		0	0.07	0.00	-0.02
**9**	8/8	Pass		0	-0.04	0.18	0.14
**10**	8/8	Pass		0	-0.02	0.06	0.03
**11**	8/8	Pass		0	0.08	0.17	0.12
**12**	8/8	Pass		0	-0.17	0.01	-0.11
**13**	8/8	Pass		0	0.06	0.05	0.11
			**Mean**	**0**	**-0.08**	**0.04**	**-0.05**
			**Range**	**0, 0**	**-0.36, 0.08**	**-0.07, 0.16**	**-0.21, 0.06**
			**Standard Deviation**	**0**	**0.11**	**0.07**	**0.08**
**1**	8/8	Pass		0	-0.05	-0.02	-0.01
**EQA Round 2 2018**							
**2**	8/8	Pass		0	0.10	0.14	0.15
**3**	8/8	Pass		0	-0.10	-0.02	0.01
**4**	8/8	Pass		0	-0.07	0.06	0.05
**5**	7/8	Acceptable	Loading error	0	-0.10	0.02	ERR-2096
**6**	8/8	Pass		0	-0.07	0.02	0.04
**7**	8/8	Pass		0	0.16	0.04	0.01
**8**	8/8	Pass		0	-0.19	-0.06	0.02
**9**	8/8	Pass		0	-0.26	-0.01	-0.03
**10**	8/8	Pass		0	-0.14	0.06	0.08
**11**	8/8	Pass		0	-0.13	-0.07	0.04
**12**	8/8	Pass		0	0.06	0.07	0.10
**13**	8/8	Pass		0	-0.12	0.06	0.00
			**Mean**	**0**	**-0.07**	**0.03**	**0.03**
			**Range**	**0, 0**	**-0.26, 0.16**	**-0.07, 0.17**	**-0.03, 0.15**
			**Standard Deviation**	**0**	**0.12**	**0.07**	**0.05**
